# Acute Stroke Care for Intracranial Artery Occlusion in an Aging Rural Region: Insights From the Fukushima Large Vessel Occlusion (LVO) Stroke Registry

**DOI:** 10.7759/cureus.109234

**Published:** 2026-05-19

**Authors:** Takao Kojima, Takuya Maeda, Yuhei Ito, Haruhiko Kikuta, Kiyoshi Saito, Masazumi Fujii

**Affiliations:** 1 Department of Neurosurgery, Fukushima Medical University, Fukushima, JPN; 2 Department of Neurosurgery, Chubu Rosai Hospital, Nagoya, JPN; 3 Department of Neurosurgery, Japanese Red Cross Fukushima Hospital, Fukushima, JPN; 4 Department of Neurosurgery, Hoshi General Hospital, Fukushima, JPN; 5 Department of Neurosurgery, Fukushima Rosai Hospital, Iwaki, JPN

**Keywords:** acute intracranial artery occlusion, hybrid stroke care system, japanese geriatrics, limited medical resources, mechanical thrombectomy, region-specific stroke network, treatment pathways

## Abstract

Background: Establishing an effective stroke care system for patients with acute intracranial artery occlusion is particularly challenging in regions affected by aging and depopulation with limited medical resources. We conducted a study, termed the Fukushima Large Vessel Occlusion (LVO) Stroke Registry, to clarify real-world treatment practices and patient pathways in such a setting.

Methods: This prospective multicenter registry enrolled consecutive patients with acute intracranial artery occlusion admitted within 24 h of symptom onset across 12 hospitals in Fukushima Prefecture between July 2020 and June 2022. Patients receiving mechanical thrombectomy or medical treatment were analyzed. Functional independence was defined as a modified Rankin Scale (mRS) score of 0-2 on Day 90. Among patients undergoing mechanical thrombectomy, the treatment pathways were classified as mothership, patient transfer, or physician transfer.

Results: A total of 210 patients were enrolled in this study. The median age was 81 years, and the median National Institutes of Health Stroke Scale (NIHSS) score was 18. Functional independence at 90 days was achieved in 68 patients (32.4%), while symptomatic intracranial hemorrhage occurred in 7 (3.3%), and 90-day mortality occurred in 53 (25.2%). Among 147 patients treated with mechanical thrombectomy, successful reperfusion (defined by a modified Thrombolysis in Cerebral Infarction (mTICI) 2b or 3) was achieved in 107 (72.8%). Functional independence was associated with younger age (76.5 vs. 82 years, *P* = 0.016), premorbid mRS score 0-2 (98.0% vs. 88.7%, *P *= 0.042), mothership pathway (60.0% vs. 38.1%, *P* = 0.012), lower NIHSS score (16 vs. 21, *P* < 0.001), higher Alberta Stroke Program Early CT Score (ASPECTS) (10 vs. 8, *P* < 0.001), shorter puncture-to-recanalization time (38 vs. 67 minutes, *P* < 0.001), and higher successful reperfusion rate (92.0% vs. 62.9%, *P* < 0.001). In multivariate analysis, age (odds ratio (OR) 0.959, 95% confidence interval (CI) 0.925-0.995; *P* = 0.024), NIHSS score (OR 0.914, 95% CI 0.864-0.968; *P* = 0.002), ASPECTS (OR 1.519, 95% CI 1.146-2.013; *P* = 0.004), and mTICI 2b or 3 (OR 8.741, 95% CI 2.581-29.597; *P* < 0.001) were independently associated with functional independence. Although unadjusted functional independence differed among the pathways, no clear differences in safety outcomes were observed.

Conclusions: In aging and depopulated regions, a hybrid stroke care system integrating mothership, patient transfer, and physician transfer pathways can maintain patients’ access to mechanical thrombectomy with acceptable safety. These findings highlight the importance of a flexible region-specific stroke network and the need for workflow optimization to improve clinical outcomes in resource-limited settings.

## Introduction

Mechanical thrombectomy is an established treatment for acute ischemic stroke caused by large-vessel occlusion. Following landmark randomized trials [1-9] and subsequent guideline updates [10,11], endovascular therapy has been widely adopted as the primary treatment strategy, achieving substantial improvements in clinical outcomes in appropriately selected patients. In recent years, the scope of endovascular treatment has expanded beyond classic large-vessel occlusion to include selected cases of medium-vessel occlusion [12] and posterior circulation stroke [13]. Consequently, acute stroke care increasingly encompasses a broader spectrum of intracranial artery occlusions, requiring flexible treatment strategies tailored to patient characteristics and regional healthcare resources.

However, the organization and delivery of acute stroke care may vary substantially among aging rural areas. In such settings, multiple factors may influence whether mechanical thrombectomy can be offered in a timely manner, including patient access to centers capable of performing mechanical thrombectomy, availability of physicians trained in endovascular treatment, transport distance, and hospital capacity. In addition, treatment decisions remain particularly challenging for patients in the gray-zone categories, including medium- and distal-vessel occlusion, posterior circulation occlusion, mild neurological deficits, and extensive early ischemic changes. In these situations, management often depends not only on clinical evidence but also on local expertise and available resources.

These issues are particularly relevant in Fukushima Prefecture, a non-metropolitan region of Japan with an aging population, progressive depopulation, and a geographically dispersed healthcare system. Therefore, to clarify the real-world implementation of acute stroke care for intracranial artery occlusion in this setting, we conducted the Fukushima Large Vessel Occlusion (LVO) Stroke Registry, a prospective multicenter observational study involving stroke centers across the prefecture. This registry was designed to capture the actual treatment patterns, including medical management and mechanical thrombectomy, and to evaluate the associations between treatment selection, care pathways, and clinical outcomes.

In the present study, we analyzed a prospective Fukushima LVO Stroke Registry cohort to evaluate real-world acute stroke care for intracranial artery occlusion in an aging rural region and to examine the association between treatment pathways and clinical outcomes. In addition, we aimed to assess whether a hybrid regional stroke system integrating mothership, patient transfer, and physician transfer pathways could maintain access to mechanical thrombectomy with acceptable safety in a resource-limited setting.

## Materials and methods

Study design and setting

This study used data from the Fukushima LVO Stroke Registry, a prospective multicenter observational registry conducted in Fukushima Prefecture, Japan. The registry enrolled patients admitted to 12 participating hospitals, including primary stroke and mechanical thrombectomy-capable centers, between July 2020 and June 2022. This study thus aimed to capture the real-world status of acute stroke care using a regional medical system. Although originally named the Fukushima LVO Stroke Registry, it prospectively enrolled patients with acute intracranial artery occlusion, including selected medium-vessel and posterior circulation occlusions. The study protocol was approved by the Ethics Committee of the Fukushima Medical University (approval number 2020-050). Patients were prospectively registered after being informed of study participation and providing consent in accordance with the registry protocol.

Patient selection

We analyzed consecutively treated adult patients aged 18 years or older who were admitted within 24 h of stroke onset (or the time last known well) and diagnosed with acute intracranial artery occlusion using computed tomography angiography (CTA), magnetic resonance angiography (MRA), or digital subtraction angiography (DSA). All registered patients with acute intracranial artery occlusion during the study period were screened for inclusion. Patients with incomplete data were excluded from the final analysis, and no imputation methods were applied for missing variables.

Treatment decisions regarding mechanical thrombectomy, including device selection and procedural management, were made at the discretion of the treating neuroendovascular specialists at each institution according to contemporary Japanese guidelines [11].

Treatment pathway classification

To analyze care delivery in patients who underwent mechanical thrombectomy, the treatment pathways were classified into three categories: mothership, patient transfer, and physician transfer. *Mothership* refers to direct admission to a mechanical thrombectomy-capable center. *Patient transfer* refers to the interhospital transfer of a patient from the initial hospital to another for endovascular treatment. *Physician transfer* refers to the transfer of a neuroendovascular specialist to the receiving hospital for mechanical thrombectomy.

Collected variables and imaging assessment

Baseline characteristics, including age, sex, cerebrovascular risk factors and comorbidities, laboratory findings, premorbid modified Rankin Scale (mRS) score [14], National Institutes of Health Stroke Scale (NIHSS) score on admission [15], Alberta Stroke Program Early CT Score (ASPECTS) [16], location of intracranial artery occlusion, stroke subtype, and use of intravenous recombinant tissue plasminogen activator (rt-PA), were collected.

Imaging interpretation was performed locally at each participating institution as part of routine clinical practice. No central imaging core laboratory, blinded image review, or formal inter-rater reliability assessment was conducted.

Because diagnostic modalities differ across institutions, patients who underwent MRI also underwent ASPECTS, measured on diffusion-weighted imaging (DWI). In patients with posterior circulation stroke, posterior circulation ASPECTS (pc-ASPECTS) was measured using DWI. In the present study, ASPECTS or pc-ASPECTS was defined as the ASPECTS on non-contrast CT, DWI, or DWI pc-ASPECTS. Occlusion sites were assessed using CTA, MRA, or DSA and categorized according to location.

In patients who underwent mechanical thrombectomy, procedural variables included the first-line thrombectomy technique, puncture time, reperfusion grade, and workflow metrics, such as onset-to-door, door-to-imaging, imaging-to-puncture, puncture-to-recanalization, and onset-to-recanalization times. First-line thrombectomy devices were used during the initial attempt to recanalize the vessel, and included a stent retriever, an aspiration catheter, or both (combined technique). Reperfusion following mechanical thrombectomy was graded using the modified Thrombolysis in Cerebral Infarction (mTICI) scale [17].

Intracranial hemorrhage within 72 hours of onset was assessed. Symptomatic intracranial hemorrhage was defined as a worsening of four or more points on the NIHSS score from baseline or death [18]. Functional outcomes at 90 days were assessed using the mRS score during outpatient visits or telephone follow-up at each participating institution.

Outcomes

The primary outcomes were functional independence 90 days after stroke onset and symptomatic intracranial hemorrhage. Functional independence was defined as an mRS score of 0 to 2. Secondary outcomes included 90-day mortality among patients treated with mechanical thrombectomy, successful reperfusion, and the time from onset or last known well to recanalization. Successful reperfusion was defined as mTICI score of 2b or 3.

Statistical analysis

Continuous variables were summarized as medians with interquartile ranges and compared using the Mann-Whitney U or Kruskal-Wallis test, as appropriate. Categorical variables are summarized as counts and percentages and were compared using the Chi-square test or Fisher’s exact test. Multivariate logistic regression analysis was conducted to identify the independent predictors of functional independence. Variables entered into the model included age, premorbid mRS score of 0-2, admission NIHSS score, ASPECTS/pc-ASPECTS, onset-to-recanalization time, and successful reperfusion based on univariate significance. Statistical significance was set at *P* < 0.05. Statistical analyses were performed using IBM SPSS Statistics version 31 (IBM Corp., Armonk, NY).

## Results

Patient cohort

**Figure 1:**
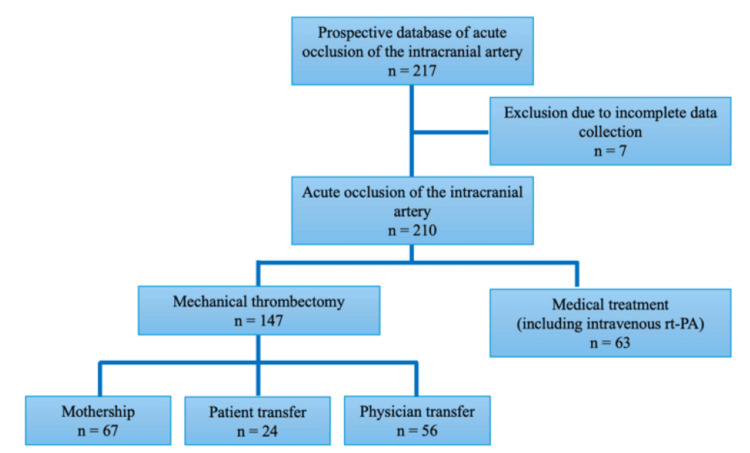
Flow diagram of patient selection. A total of 217 patients were registered, of whom 210 were included in the final analysis after excluding incomplete cases. Among them, 147 (70%) underwent mechanical thrombectomy, whereas 63 (30%) received medical treatment. Patients who underwent mechanical thrombectomy were categorized into three treatment pathways: mothership (n = 67, 45.6%), patient transfer (n = 24, 16.3%), and physician transfer (n = 56, 38.1%). Image credit: All authors.

A total of 217 patients were registered, of whom 210 were included in the final analysis after exclusion of incomplete cases. Among them, 147 (70.0%) underwent mechanical thrombectomy, whereas 63 (30.0%) received medical treatment, including intravenous thrombolysis. Patients who underwent mechanical thrombectomy were categorized into three treatment pathways: mothership (*n* = 67, 45.6%), patient transfer (*n* = 24, 16.3%), and physician transfer (*n* = 56, 38.1%) (Figure 1).

Baseline characteristics and overall outcomes

The baseline characteristics and clinical outcomes of the study cohort are summarized in Table 1. The median age was 81 years (interquartile range (IQR), 73-88 years), and 111 patients (52.9%) were male. Premorbid functional independence was observed in 175 patients (83.3%). The median NIHSS score on admission was 18 (IQR, 13-24), and the median ASPECTS or pc-ASPECTS was 9 (IQR, 7-10). The most common site of occlusion was the M1 segment of the middle cerebral artery (43.8%), followed by the intracranial internal carotid artery (14.8%). Cardioembolism was the predominant stroke subtype, observed in 72.9% of patients. Intravenous rt-PA was administered to 99 patients (47.1%), and mechanical thrombectomy was performed in 147 (70.0%). Workflow metrics showed a median onset-to-door time of 88.5 minutes (IQR, 50-220 minutes) and door-to-imaging time of 40 minutes (IQR, 28-59 minutes). Among patients treated with mechanical thrombectomy, the median puncture-to-recanalization time was 58.5 minutes (IQR, 36-84 minutes), while the median onset-to-recanalization time was 267 minutes (IQR, 201-372.5 minutes). Successful reperfusion was achieved in 107 (72.8 %) patients. Intracranial hemorrhage within 72 h occurred in 37 patients (17.6%), including symptomatic intracranial hemorrhage in seven patients (3.3%). At 90 days, functional independence (mRS 0-2) was observed in 68 patients (32.4%), and the 90-day mortality rate was 25.2%.

**Table 1:** Baseline characteristics and overall outcomes of the study population. A summary of the demographic, clinical, radiological, and workflow metrics and outcomes of all included patients (*n* = 210) is presented. mRS, modified Rankin Scale; NIHSS, National Institutes of Health Stroke Scale; ASPECTS, Alberta Stroke Program Early CT Score; pc-ASPECTS, posterior circulation Alberta Stroke Program Early CT Score; ICA, internal carotid artery; MCA, middle cerebral artery; ACA, anterior cerebral artery; BA, basilar artery; VA, vertebral artery; PCA, posterior cerebral artery; rt-PA, recombinant tissue plasminogen activator; mTICI, modified Thrombolysis in Cerebral Infarction

Variable	*n* = 210
Age, median (IQR), years	81 (73-88)
Male sex, *n* (%)	111 (52.9)
Hypertension, *n* (%)	150 (71.4)
Dyslipidemia, *n* (%)	45 (21.4)
Diabetes mellitus, *n* (%)	40 (19.0)
Atrial fibrillation, *n* (%)	97 (46.2)
History of ischemic stroke, *n* (%)	42 (20.0)
Current smoker, *n* (%)	38 (18.1)
in-hospital stroke onset, *n* (%)	27 (12.9)
mRS score 0-2 premorbid, *n* (%)	175 (83.3)
NIHSS on admission, median (IQR)	18 (13-24)
ASPECTS or pc-ASPECTS, median (IQR)	9 (7-10)
Location of vessel occlusion	
Extracranial ICA, *n* (%)	14 (6.7)
Intracranial ICA, *n* (%)	31 (14.8)
MCA M1 segment, *n* (%)	92 (43.8)
MCA M2 segment and beyond, *n* (%)	39 (18.6)
ACA, *n* (%)	3 (1.4)
BA, *n* (%)	14 (6.7)
VA, *n* (%)	2 (1.0)
PCA, *n* (%)	1 (0.5)
Multi-vessel occlusion in anterior and posterior circulation, *n* (%)	14 (6.7)
Stroke classification	
Cardioembolism, *n* (%)	153 (72.9)
Large artery atherosclerosis, *n* (%)	32 (15.2)
Other determined cause, *n* (%)	12 (5.7)
Undetermined cause, *n* (%)	13 (6.2)
Intravenous rt-PA use, *n* (%)	99 (47.1)
Mechanical thrombectomy, *n* (%)	147 (70.0)
Onset to door, median (IQR), minutes	88.5 (50-220)
Door to imaging, median (IQR), minutes	40 (28-59)
Imaging to needle, median (IQR), minutes	28 (18.5-38.5)
Imaging to puncture, median (IQR), minutes	49 (34-74)
Puncture to recanalization, median (IQR), minutes	58.5 (36-84)
Onset to recanalization, median (IQR), minutes	267 (201-372.5)
mTICI 2b or 3, *n* (%)	107 (72.8)
Intracranial hemorrhage within 72 hours, *n* (%)	37 (17.6)
Symptomatic intracranial hemorrhage within 72 hours, *n* (%)	7 (3.3)
mRS score at 90 days	
mRS 0-2, *n* (%)	68 (32.4)
mRS 0, *n* (%)	19 (9.0)
mRS 1, *n* (%)	21 (10.0)
mRS 2, *n* (%)	28 (13.3)
mRS 3, *n* (%)	18 (8.6)
mRS 4, *n* (%)	28 (13.3)
mRS 5, *n* (%)	43 (20.5)
mRS 6, *n* (%)	53 (25.2)

Factors associated with functional outcomes in the mechanical thrombectomy cohort

To identify factors associated with clinical outcomes, patients who underwent mechanical thrombectomy were divided into two groups according to their 90-day functional outcomes: favorable outcomes (mRS 0-2, *n* = 50) and unfavorable outcomes (mRS 3-6, *n* = 97). Baseline characteristics and clinical variables according to outcome are presented in Table 2. In the univariate analysis, favorable outcomes were associated with younger age (76.5 vs. 82 years, *P* = 0.016), premorbid mRS score 0-2 (98.0% vs. 88.7%, *P* = 0.042), lower NIHSS score (median 16 vs. 21, *P* < 0.001), and higher ASPECTS or pc-ASPECTS (median 10 vs. 8, *P* < 0.001). In addition, the mothership pathway was more common in the favorable outcome group (60.0% vs. 38.1%, *P* = 0.012), while successful reperfusion was significantly higher (92.0% vs. 62.9%, *P* < 0.001). The procedural time was also shorter in the favorable outcome group, including the puncture-to-recanalization time (median 38 vs. 67 minutes, *P* < 0.001) and onset-to-recanalization time (median 224.5 vs. 289 minutes, *P* = 0.039). Variables with *P* < 0.05 in univariate analysis were entered into a multivariate logistic regression model. In multivariate analysis, age (OR 0.959, 95% CI, 0.925-0.995; *P* = 0.024), NIHSS score (OR 0.914, 95% CI, 0.864-0.968; *P* = 0.002), ASPECTS (OR 1.519, 95% CI, 1.146-2.013; *P* = 0.004), and mTICI 2b or 3 (OR 8.741, 95% CI 2.581-29.597; *P* < 0.001) were all found to be independently associated with functional outcome (Table 3).

**Table 2:** Comparison of baseline characteristics and outcomes between mechanical thrombectomy patients with favorable and unfavorable outcomes. The patients were divided into favorable (mRS 0–2) and unfavorable (mRS 3–6) functional outcome groups based on mRS scores. mRS, modified Rankin Scale; NIHSS, National Institutes of Health Stroke Scale; ASPECTS, Alberta Stroke Program Early CT Score; pc-ASPECTS, posterior circulation Alberta Stroke Program Early CT Score; ICA, internal carotid artery; MCA, middle cerebral artery; ACA, anterior cerebral artery; BA, basilar artery; VA, vertebral artery; rt-PA, recombinant tissue plasminogen activator; mTICI, modified Thrombolysis in Cerebral Infarction

Variable	mRS 0-2 (*n* = 50)	mRS 3-6 (*n* = 97)	*P*-value
Age, median (IQR), years	76.5 (69-84)	82 (75-87)	0.016
Male sex, *n* (%)	30 (60.0)	50 (51.5)	0.330
Hypertension, *n* (%)	35 (70.0)	67 (69.1)	0.908
Dyslipidemia, *n* (%)	13 (26.0)	18 (18.6)	0.295
Diabetes mellitus, *n* (%)	11 (22.0)	14 (14.4)	0.247
Atrial fibrillation, *n* (%)	22 (44.0)	48 (49.5)	0.528
History of ischemic stroke, *n* (%)	8 (16.0)	20 (20.6)	0.499
Current smoker, *n* (%)	14 (29.2)	16 (17.4)	0.107
in-hospital stroke onset, *n* (%)	5 (10.0)	12 (12.4)	0.670
mRS score 0-2 premorbid, *n* (%)	49 (98.0)	86 (88.7)	0.042
Mothership, *n* (%)	30 (60.0)	37 (38.1)	0.012
NIHSS on admission, median (IQR)	16 (12-20)	21 (16-27)	<0.001
ASPECTS or pc-ASPECTS, median (IQR)	10 (9-10)	8 (7-10)	<0.001
Location of vessel occlusion			0.684
Extracranial ICA, *n* (%)	2 (4.0)	4 (4.1)	
Intracranial ICA, *n* (%)	10 (20.0)	21 (21.6)	
MCA M1 segment, *n* (%)	27 (54.0)	43 (44.3)	
MCA M2 segment and beyond, *n* (%)	5 (10.0)	10 (10.3)	
ACA, *n* (%)	0 (0.0)	2 (2.1)	
BA, *n* (%)	4 (8.0)	5 (5.2)	
VA, *n* (%)	0 (0.0)	2 (2.1)	
Multi-vessel occlusion in anterior and posterior circulation, *n* (%)	2 (4.0)	10 (10.3)	
Stroke classification			0.920
Cardioembolism, *n* (%)	36 (72.0)	71 (73.2)	
Large artery atherosclerosis, *n* (%)	6 (12.0)	13 (13.4)	
Other determined cause, *n* (%)	4 (8.0)	8 (8.2)	
Undetermined cause, *n* (%)	4 (8.0)	5 (5.2)	
Intravenous rt-PA use, *n* (%)	24 (48.0)	50 (51.5)	0.684
First-line devices for mechanical thrombectomy			
Combined technique, *n* (%)	31 (62.0)	71 (73.2)	
Stent retriever, *n* (%)	3 (6.0)	7 (7.2)	
Aspiration catheter, *n* (%)	13 (26.0)	9 (9.3)	
Other, *n* (%)	3 (6.0)	4 (4.1)	
Attempt, *n* (%)	0 (0.0)	6 (6.2)	
Onset to door, median (IQR), minutes	74 (49-135)	75 (50-164)	0.859
Door to imaging, median (IQR), minutes	41.5 (23-65)	39 (23-53)	0.314
Imaging to needle, median (IQR), minutes	26 (18-34.5)	28.5 (17-38)	0.759
Imaging to puncture, median (IQR), minutes	48 (32-64)	51 (35-82)	0.279
Puncture to recanalization, median (IQR), minutes	38 (28-65)	67 (44-90)	<0.001
Onset to recanalization, median (IQR), minutes	224.5 (179-368)	289 (225-377)	0.039
mTICI 2b or 3, *n* (%)	46 (92.0)	61 (62.9)	<0.001
Intracranial hemorrhage within 72 hours, *n* (%)	6 (12.0)	24 (24.7)	0.101
Symptomatic intracranial hemorrhage within 72 hours, *n* (%)	0 (0.0)	5 (5.2)	0.150

**Table 3:** Multivariate logistic regression analysis for predictors of favorable outcomes in patients who underwent mechanical thrombectomy. Variables with p < 0.05 in univariate analysis were included in the multivariate model. mRS, modified Rankin Scale; NIHSS, National Institutes of Health Stroke Scale; ASPECTS, Alberta Stroke Program Early CT Score; pc-ASPECTS, posterior circulation Alberta Stroke Program Early CT Score; mTICI, modified Thrombolysis in Cerebral Infarction

Variable	Odds ratio (95% CI)	*P*-value
Age	0.959 (0.925-0.995)	0.024
mRS score 0-2 premorbid	4.655 (0.540-40.155)	0.162
NIHSS on admission	0.914 (0.864-0.968)	0.002
ASPECTS or pc-ASPECTS	1.519 (1.146-2.013)	0.004
Onset to recanalization	1.000 (0.998-1.002)	0.971
mTICI 2b or 3	8.741 (2.581-29.597)	<0.001

Sub-analysis according to treatment pathway

A sub-analysis was conducted among patients who underwent mechanical thrombectomy to evaluate the impact of the treatment pathway (mothership, patient transfer, and physician transfer). Baseline characteristics and outcomes according to pathway are presented in Table 4. Age differed significantly between the three groups (median 80, 74, and 83 years for mothership, patient transfer, and physician transfer, respectively; *P* = 0.008). Male sex and the prevalence of diabetes mellitus also differed significantly between the groups (*P* = 0.004 and *P* = 0.013, respectively). Workflow metrics varied according to pathway. The imaging-to-puncture time was significantly shorter in the mothership group (median, 40 minutes) than in the patient transfer (89.5 minutes) and physician transfer (50 minutes) groups (*P* < 0.001). The puncture-to-recanalization time was also shortest in the mothership group (49 minutes vs. 63.5 and 70.5 minutes; *P* = 0.021). The clinical outcomes differed across pathways. Functional independence at 90 days was significantly more common in the mothership group (30, 44.8%) than in the patient transfer (6, 25.0%) or physician transfer (14, 25.0%) groups (*P* = 0.042). In contrast, no significant differences in intracranial hemorrhage, symptomatic intracranial hemorrhage, or mortality were identified among the three groups.

**Table 4:** Comparison of baseline characteristics and outcomes among different patient pathways. Patients were categorized into the mothership, patient transfer, and physician transfer groups. mRS, modified Rankin Scale; NIHSS, National Institutes of Health Stroke Scale; ASPECTS, Alberta Stroke Program Early CT Score; pc-ASPECTS, posterior circulation Alberta Stroke Program Early CT Score; ICA, internal carotid artery; MCA, middle cerebral artery; ACA, anterior cerebral artery; BA, basilar artery; VA, vertebral artery; rt-PA, recombinant tissue plasminogen activator; mTICI, modified Thrombolysis in Cerebral Infarction

Variable	Mothership (*n* = 67)	Patient transfer (*n* = 24)	Physician transfer (*n* = 56)	*P*-value
Age, median (IQR), years	80 (73.5-86)	74 (69.5-79.5)	83 (76-89.5)	0.008
Male sex, *n* (%)	36 (53.7)	20 (83.3)	24 (42.9)	0.004
Hypertension, *n* (%)	49 (73.1)	14 (58.3)	39 (69.6)	0.401
Dyslipidemia, *n* (%)	17 (25.4)	3 (12.5)	11 (19.6)	0.392
Diabetes mellitus, *n* (%)	16 (23.9)	6 (25.0)	3 (5.4)	0.013
Atrial fibrillation, *n* (%)	34 (50.7)	8 (33.3)	28 (50.0)	0.308
History of ischemic stroke, *n* (%)	12 (17.9)	6 (25.0)	10 (17.9)	0.719
Current smoker, *n* (%)	13 (20.6)	5 (21.7)	12 (22.2)	0.978
mRS score 0-2 premorbid, *n* (%)	63 (94.0)	24 (100.0)	48 (85.7)	0.068
NIHSS on admission, median (IQR)	17 (12-21.5)	19 (15-23.5)	20.5 (15.5-27)	0.094
ASPECTS or pc-ASPECTS, median (IQR)	9 (7.5-10)	9 (7.5-10)	9 (7-10)	0.798
Location of vessel occlusion				0.269
Extracranial ICA, *n* (%)	3 (4.5)	2 (8.3)	1 (1.8)	
Intracranial ICA, *n* (%)	14 (20.9)	4 (16.7)	13 (23.2)	
MCA M1 segment, *n* (%)	30 (44.8)	9 (37.5)	31 (55.4)	
MCA M2 segment and beyond, *n* (%)	9 (13.4)	4 (16.7)	2 (10.7)	
ACA, *n* (%)	2 (3.0)	0 (0.0)	0 (0.0)	
BA, *n* (%)	6 (9.0)	1 (4.2)	2 (3.6)	
VA, *n* (%)*n* (%)	1 (1.5)	0 (0.0)	1 (1.8)	
Multi-vessel occlusion in anterior and posterior circulation, *n* (%)	2 (3.0)	4 (16.7)	6 (10.7)	
Stroke classification				0.239
Cardioembolism, *n* (%)	50 (74.6)	14 (58.3)	43 (76.8)	
Large artery atherosclerosis, *n* (%)	6 (9.0)	7 (29.2)	6 (10.7)	
Other determined cause, *n* (%)	7 (10.4)	1 (4.2)	4 (7.1)	
Undetermined cause, *n* (%)	4 (6.0)	2 (8.3)	3 (5.4)	
Intravenous rt-PA use, *n* (%)	32 (47.8)	16 (66.7)	26 (46.4)	0.214
First-line devices for mechanical thrombectomy				0.564
Combined technique, *n* (%)	47 (70.1)	16 (66.7)	39 (69.6)	
Stent retriever, *n* (%)	4 (6.0)	1 (4.2)	5 (8.9)	
Aspiration catheter, *n* (%)	12 (17.9)	2 (8.3)	8 (14.3)	
Other, *n* (%)	2 (3.0)	3 (12.5)	2 (3.6)	
Attempt, *n* (%)	2 (3.0)	2 (8.3)	2 (3.6)	
Onset to door, median (IQR), minutes	88 (49.5-211)	60 (41.5-96)	75 (51-189)	0.167
Door to imaging, median (IQR), minutes	39 (23-63.5)	43 (38-53.5)	36 (21.5-56.5)	0.410
Imaging to needle, median (IQR), minutes	20 (10-34)	24.5 (14.5-33)	34 (23-49)	0.024
Imaging to puncture, median (IQR), minutes	40 (24.5-52)	89.5 (75-119.5)	50 (40.5-62.5)	<0.001
Puncture to recanalization, median (IQR), minutes	49 (31-79)	63.5 (38-81)	70.5 (40.5-98)	0.021
Onset to recanalization, median (IQR), minutes	232 (178-358)	291 (245-357.5)	297 (219-415)	0.101
mTICI 2b or 3, *n* (%)	55 (82.1)	17 (70.8)	35 (62.5)	0.051
Intracranial hemorrhage within 72 hours, *n* (%)	11 (16.4)	6 (25.0)	13 (23.2)	0.320
Symptomatic intracranial hemorrhage within 72 hours, *n* (%)	1 (1.5)	2 (8.3)	2 (3.6)	0.212
mRS score at 90 days				
mRS 0-2, *n* (%)	30 (44.8)	6 (25.0)	14 (25.0)	0.042
mRS 0, *n* (%)	10 (14.9)	0 (0.0)	3 (5.4)	
mRS 1, *n* (%)	8 (11.9)	2 (8.3)	6 (10.7)	
mRS 2, *n* (%)	12 (17.9)	4 (16.7)	5 (8.9)	
mRS 3, *n* (%)	5 (7.5)	3 (12.5)	2 (3.6)	
mRS 4, *n* (%)	6 (9.0)	6 (25.0)	10 (17.9)	
mRS 5, *n* (%)	12 (17.9)	4 (16.7)	17 (30.4)	
mRS 6, *n* (%)	14 (20.9)	5 (20.8)	13 (23.2)	0.946

## Discussion

Overall, this study provides two key insights into acute stroke care in aging and rural regions. First, it clarifies the real-world implementation of acute stroke treatment, demonstrating that mechanical thrombectomy can be widely performed, even in a resource-limited setting. Second, multiple patient pathways can be used in a complementary manner to deliver timely treatment.

The implications of our findings for regional stroke systems in Japan should also be considered in light of the important differences in study settings. Previous Japanese registries, including the RESCUE-Japan Registry 2 [19] and the K-NET Registry [20], have provided valuable real-world evidence regarding endovascular therapy for acute large-vessel occlusion; however, they reflect nationwide or metropolitan practice rather than the experience of an aging, depopulating rural region. In addition, the Tama registry from suburban Tokyo describes real-world mechanical thrombectomy practices in a densely populated urban area and was presented as reflecting an emergent transfer system in that setting [21]. Therefore, the present results from the Fukushima LVO Stroke Registry should not be interpreted simply as another estimate of mechanical thrombectomy outcomes in Japan, nor should they be directly compared with nationwide or urban registries without careful consideration of the regional context. Instead, the principal significance of this study lies in demonstrating how LVO care is organized and delivered in a region characterized by population aging, depopulation, geographic dispersion, and an uneven distribution of medical resources, as well as the levels of treatment performance and clinical outcomes that can be achieved under these conditions. This context-specific perspective may be particularly important when designing sustainable stroke care systems in super-aging rural areas. In the mechanical thrombectomy cohort, favorable outcomes were found to be associated with a younger age, lower baseline stroke severity, higher ASPECTS or pc-ASPECTS scores, shorter puncture-to-recanalization time, and a higher rate of successful reperfusion. These findings are clinically plausible and underscore the importance of pretreatment conditions and procedural efficiency. In particular, the association between shorter puncture-to-recanalization time and better outcomes indicates that even after the decision to pursue mechanical thrombectomy has been made, events within the endovascular workflow remain highly relevant to eventual recovery.

Currently, the optimal organization of stroke pathways remains controversial. The REVASCAT trial and subsequent studies emphasized the importance of ensuring rapid access to centers capable of performing mechanical thrombectomy [22]. Observational studies, including those by Morey et al., have further compared direct transport and interhospital transfer strategies, highlighting the tradeoffs between early thrombolysis and timely mechanical thrombectomy [23]. Simulation-based analyses by Maas et al. demonstrated that no single pathway is universally optimal, and that regional characteristics strongly influence the effectiveness of different models [24,25]. More recently, Hubert et al. reported that physician transfer (flying intervention teams) could reduce treatment delays in rural settings, although its implementation depended on available resources and infrastructure [26]. Notably, our study also demonstrated that a hybrid system incorporating mothership, patient transfer, and physician transfer pathways could be effectively implemented in rural regions. Each pathway showed distinct advantages and disadvantages [27,28]. The mothership pathway may offer the most straightforward route to rapid mechanical thrombectomy when direct access to a mechanical thrombectomy-capable center is feasible. Patient transfer may further help to preserve timely initial evaluation and intravenous thrombolysis at the nearest stroke center; however, it remains vulnerable to door-in-door-out delays and interhospital transport time [29]. Physician transfer may additionally reduce EVT delays while preserving local access; however, it is logistically demanding and depends on institutional preparedness [30]. These findings indicate that the optimal pathway is context dependent, and that pathway comparisons should be interpreted cautiously because pathway allocation was non-randomized and baseline differences existed among the groups. In a prefecture such as Fukushima, where geography, transport distance, and specialist availability vary across sub-regions, the practical goal is therefore unlikely to rely exclusively on a single pathway. Instead, the present data support the continued refinement of a flexible regional network that combines mothership, patient transfer, and physician transfer, according to local circumstances. Therefore, the overall safety findings of this study should be interpreted with caution. Although intracranial hemorrhage was more common in the mechanical thrombectomy group, symptomatic intracranial hemorrhage was infrequent, while hemorrhagic complications did not differ significantly across the treatment pathways. These findings further support the feasibility of implementing broad mechanical thrombectomy within a coordinated regional network, even in aging rural populations.

This study had several limitations. First, it was an observational registry study, and the treatment allocation was not randomized. The inclusion of anterior circulation, posterior circulation, and selected medium-vessel occlusions may have introduced additional clinical heterogeneity. In addition, inter-center variability in thrombectomy expertise, imaging interpretation, procedural management, and workflow protocols may have influenced treatment selection and clinical outcomes. Second, the mechanical thrombectomy and medical treatment groups differed substantially in terms of baseline characteristics, particularly age, premorbid functional status, and stroke severity, thus limiting the causal interpretation of between-group outcome differences. Third, pathway analysis was secondary and remained susceptible to confounding factors among the three pathway groups. Comparisons among treatment pathways may have been affected by geographic, demographic, and baseline clinical differences among patient groups, including differences in age and frailty. Fourth, the sample size was modest, particularly in the patient transfer group; therefore, some comparisons may have been underpowered. Fifth, this registry reflects the experiences of only a single prefecture, and the generalizability of the findings to other regions should therefore be interpreted with caution. Finally, the study period overlapped with the COVID-19 pandemic, which may have influenced the transfer logistics, hospital resource availability, and acute stroke workflow. Thus, overall, caution should be exercised when generalizing these findings to non-pandemic settings.

## Conclusions

In this prospective multicenter registry from an aging rural prefecture in Japan, mechanical thrombectomy was performed in a substantial proportion of patients with acute intracranial artery occlusion, with acceptable reperfusion and safety outcomes observed in real-world practice. A regional stroke system incorporating mothership, patient transfer, and physician transfer pathways may facilitate the real-world implementation of mechanical thrombectomy across geographically dispersed areas without apparent major safety disadvantages. The principal significance of the Fukushima LVO Stroke Registry lies in demonstrating how acute stroke care for intracranial artery occlusion is organized and delivered, as well as the level of treatment performance that can be achieved in a region characterized by population aging, depopulation, and an uneven distribution of medical resources.
